# Small cell lung cancer: from immunobiological mechanisms to clinical advances

**DOI:** 10.3389/fimmu.2026.1765497

**Published:** 2026-03-11

**Authors:** Xian Zhong, Pingping Ji, Lulu Yang

**Affiliations:** 1 Department of Medical Oncology, the Second Affiliated Hospital, Zhejiang University School of Medicine, Hangzhou, Zhejiang, China; 2 Cancer Institute (Key Laboratory of Cancer Prevention and Intervention, Chinese National Ministry of Education), the Second Affiliated Hospital, Zhejiang University School of Medicine, Hangzhou, Zhejiang, China; 3 Department of Medical Oncology, Linhai First Hospital, Taizhou, Zhejiang, China; 4 Department of Medical Oncology, Ninghai First Hospital, Ningbo, Zhejiang, China

**Keywords:** biomarker, immunosuppressive tumormicroenvironment, immunotherapy, molecular subtyping, small cell lung cancer

## Abstract

Small cell lung cancer (SCLC) remains one of the most aggressive malignancies, characterized by limited therapeutic options and persistently poor survival outcomes. This review summarizes recent advances in understanding the immunosuppressive tumor microenvironment, emerging therapeutic strategies, and biomarker-driven approaches that may enable more precise treatment. The SCLC microenvironment is dominated by suppressive immune cell populations—including regulatory T cells, tumor-associated macrophages, and myeloid-derived suppressor cells—that collectively inhibit antitumor immune responses. Integrative molecular and immunologic profiling has defined four transcription factor–driven subtypes, each exhibiting distinct immune phenotypes and differential responses to therapy. Although current immunotherapies have conferred meaningful yet modest clinical gains, combining PD-1/PD-L1 blockade with chemotherapy has improved survival in extensive-stage disease, and CTLA-4 inhibition demonstrates potential within combination regimens. Beyond immune checkpoint blockade, novel therapeutic modalities such as DLL3-targeted antibody–drug conjugates, bispecific T-cell engagers, and emerging B7-H3–directed strategies have shown encouraging activity in treatment-refractory settings. However, conventional biomarkers—including PD-L1 expression and tumor mutational burden—remain unreliable in SCLC, and the mechanisms underlying therapeutic resistance are still insufficiently understood. Future efforts should prioritize the refinement of molecular subtyping frameworks, the establishment of robust biomarker-guided patient stratification, the elucidation of resistance pathways, and the development of precision immunotherapies tailored to SCLC heterogeneity.

## 1 Introduction

### 1.1 Epidemiology of SCLC

Small cell lung cancer (SCLC) constitutes a substantial global health burden, accounting for approximately 14–15% of all lung cancer cases (1–4). Tobacco smoking is the predominant risk factor, as nearly all cases are directly associated with a smoking history (5). Clinically, SCLC is characterized by aggressive tumor biology and a high tendency to present at an advanced stage. Approximately 80-85% of patients are diagnosed with extensive-stage disease (ES-SCLC), defined by the presence of metastases beyond the ipsilateral hemithorax (6). The intrinsically aggressive biology of SCLC, characterized by rapid disease progression and high metastatic potential, severely limits therapeutic options and contributes to extremely poor outcomes. Historically, median overall survival (OS) rarely exceeds one year (7, 8), and the 5-year survival rate remains below 7%, underscoring the urgent need for more effective treatment strategies (9).

### 1.2 Traditional treatment approaches and unmet clinical needs

For nearly four decades, the therapeutic landscape for SCLC remained largely unchanged, with platinum-based chemotherapy serving as the mainstay of treatment since the 1980s (10, 11). In patients with limited-stage disease (LS-SCLC), adjuvant chemotherapy was occasionally administered following surgical resection, although only a small proportion of patients were eligible for surgery. For the majority of patients with ES- SCLC, systemic chemotherapy alone represented the standard of care (12, 13). First-line platinum-etoposide regimens achieved initial response rates exceeding 50%, as demonstrated in the control arms of pivotal phase III trials such as IMpower133 (ORR 64%) (14) and CASPIAN (ORR ~68-78%) (15). However, relapse typically occurred within months due to the rapid development of chemoresistance. Median OS remained approximately 9–11 months, and the 5−year survival rate was a dismal 5-7% (16). This pattern of initial chemosensitivity followed by early recurrence has posed a major challenge in clinical management (10, 17, 18).

Numerous strategies attempted to break this therapeutic plateau, yet none succeeded in improving long-term survival. The addition of the antiangiogenic agent bevacizumab to first-line EP was evaluated in two randomized trials. In the phase III GOIRC−AIFA study, bevacizumab improved progression−free survival (PFS) (6.7 vs. 5.7 months, p value = 0.030) but did not significantly extend median OS (9.8 vs. 8.9 months) and 1-year survival rates (25% vs 37%, hazard ratio (HR) 0.78, 95% confidence interval (CI) 0.58 to 1.06, p value = 0.113) (19). Similarly, the phase II SALUTE trial demonstrated a PFS benefit (5.5 vs. 4.4 months, HR 0.53, 95% CI 0.32-0.86) yet showed no OS improvement. In fact, a disadvantage was observed (9.4 vs. 10.9 months, HR 1.16, 95% CI 0.66-2.04).

Alternative chemotherapy backbones were also explored. A pivotal Japanese phase III study reported that irinotecan plus cisplatin (IP) conferred a survival advantage over EP (median OS 12.8 vs. 9.4 months, p value = 0.002). However, subsequent large US trials failed to confirm this superiority (20), and a meta−analysis suggested only modest benefit (21). A multicenter international randomized phase III study likewise demonstrated comparable efficacy between IP and EP (22). Thus, despite extensive investigation, EP/EC remained the global standard.

Second-line treatment options were limited. Topotecan has been the most commonly used agent for platinum-sensitive relapse, yet overall response rates have remained below 20%. Treatment attrition was significant, with only about 50% of patients receiving second-line therapy and merely 22% progressing to a third-line regimen (18). According to the National Comprehensive Cancer Network (NCCN) guidelines, re-treatment with the initial platinum-based regimen is recommended for relapses occurring more than six months after first-line therapy, whereas clinical trials are preferred for earlier relapses due to the limited efficacy of available agents (23).

Historically, numerous strategies aimed at improving outcomes, including the addition of a third agent, dose intensification, maintenance therapy, and alternating non-cross-resistant regimens, failed to confer a meaningful survival benefit over standard chemotherapy (24–26). This extended period of limited therapeutic progress was altered by the introduction of immune checkpoint inhibitors (10). A pivotal shift occurred with the landmark IMpower133 trial, which demonstrated that the addition of atezolizumab to standard chemotherapy significantly improved both OS and PFS compared to chemotherapy alone. The trial showed a median OS of 12.3 months versus 10.3 months (HR 0.70, 95% confidence interval (CI) 0.54–0.91, p value = 0.007) and a median PFS of 5.2 months versus 4.3 months (HR 0.77, 95% CI 0.62–0.96, p value = 0.02) (14, 27). This represented the first major advance in first-line therapy for ES-SCLC in decades.

Despite recent therapeutic progress, SCLC remains a difficult clinical challenge with multiple critical unmet needs (28). A central paradox lies in its initial high sensitivity to chemotherapy, followed by the rapid emergence of resistance and disease progression. Unlike non-small cell lung cancer (NSCLC), where targeted therapies have reshaped clinical outcomes, SCLC remains largely resistant to precision medicine and has long been considered difficult to drug. Most cases involve inactivating mutations in the tumor suppressor genes TP53 and RB1, a near-universal genomic feature established by comprehensive sequencing studies (4, 29–31). However, the identification of clinically validated molecular targets has been exceptionally limited, which constrains the development of effective targeted therapies (32, 33).

Recent studies have identified at least four molecular subtypes of SCLC, each exhibits distinct transcriptional profiles and biological behaviors, providing potential opportunities for therapeutic stratification (34, 35). However, significant intra-tumoral plasticity exists, with dynamic transitions between subtypes contributing to treatment adaptation and resistance (36, 37). This flexibility complicates the development of durable, subtype-specific therapies.

Immunotherapy has led to modest improvements in outcomes for SCLC, but the survival benefit is limited compared to other solid tumors (38). This is largely due to a profoundly immunosuppressive tumor microenvironment, characterized by poor T-cell infiltration, impaired antigen presentation, and enrichment of immunosuppressive cell populations. Consequently, durable responses are observed in only a small subset of patients, underscoring the need for immune-based strategies specifically tailored to the unique immune context of SCLC.

Addressing these challenges necessitates a deeper understanding of the complex biology and mechanisms underlying treatment resistance in SCLC. This review synthesizes recent advances in understanding the immunosuppressive tumor microenvironment, which has become a key barrier to effective immunotherapy, and evaluates emerging therapeutic strategies that are beginning to reshape clinical practice. Furthermore, we highlight progress in biomarker discovery, an area that has been relatively underexplored in SCLC research but holds significant promise for guiding personalized therapy. By integrating mechanistic insights with clinical relevance, this review aims to provide a comprehensive and forward-looking perspective to inform translational research and therapeutic innovation in SCLC.

## 2 Immunosuppressive landscape of SCLC

### 2.1 Transcription factor-defined subtypes

As early as the 1980s, SCLC was recognized to possess distinct histological and biological characteristics, leading to its initial classification into classic and variant subtypes (39). The classic subtype demonstrates prominent neuroendocrine (NE) features, marked by high expression of NE markers, whereas the variant subtype exhibits reduced or absent expression of these markers, reflecting non-neuroendocrine (non-NE) phenotypes (40). During this period, studies using SCLC cell lines identified differences in growth patterns, such as suspension aggregates compared to adherent monolayers, and in the expression of neuroendocrine markers including L-dopa decarboxylase and neuron-specific enolase (40–45). These findings established an early framework for distinguishing NE from non-NE phenotypes in SCLC.

However, these initial classifications were largely phenotypic and based on a limited set of molecular markers, offering minimal insight into the transcriptional regulators governing subtype identity. In 2019, Rudin et al. proposed a refined molecular taxonomy of SCLC through integrative transcriptomic analyses (34). This classification system defined four molecular subtypes—SCLC-A, SCLC-N, SCLC-P, and SCLC-Y—driven by distinct lineage-specific transcription factors: ASCL1, NEUROD1, POU2F3, and YAP1, respectively. Subsequently, in 2021, Gay et al. identified a fifth subtype, SCLC-I, characterized by low expression of canonical transcription factors and enrichment of inflammatory gene signatures (35). **Table 1** summarizes the key molecular and phenotypic features of these subtypes.

**Table 1:** Characteristics of the five molecular subtypes of small cell lung cancer (SCLC).

Subtype	Key defining marker	Prevalence	Biological characteristics	Immune microenvironment (35)	Survival (5-year OS) (52)
SCLC-A	ASCL1	~70%	Neuroendocrine	Moderate; 26% inflamed; low CD8^+^/PD-L1	Moderate (61.9%)
SCLC-N	NEUROD1	~11%	Neuroendocrine	Cold; 11% inflamed; very low CD8^+^/PD-L1	Moderate (69.3%)
SCLC-P	POU2F3	~10%	Non-neuroendocrine	Hot; 53% inflamed; high CD8^+^/PD-L1	Poor (41.7%)
SCLC-Y	YAP1	2-10%	Non-neuroendocrine	Moderate-to-hot; high CD8^+^/PD-L1	limited data
SCLC-I	ASCL1^-^/NEUROD1^-^/POU2F3^-^; Inflamed Signature	~15-17%	Non-neuroendocrine	Hot; 80-90% inflamed; high CD8^+^/PD-L1	Best (85.7%)

OS, overall survival; PD−L1, programmed death−ligand 1; CI, confidence interval. Percentages in the “Immune Microenvironment” column indicate the proportion of cases exhibiting an “inflamed” phenotype.

Among these, SCLC-A is the most prevalent, representing approximately 70% of cases. It displays classic NE features with high expression of markers such as INSM1, Delta-like ligand 3 (DLL3), BCL-2, SOX2, MYCL, E-cadherin, and TTF1, and frequently exhibits epithelial-mesenchymal transition (EMT) traits that contribute to its aggressive behavior (34, 46). SCLC-N, accounting for about 11% of cases, also demonstrates neuroendocrine differentiation but is molecularly distinct, with frequent MYC amplification and elevated expression of AURK, HES6, and INSM1. In contrast to the tightly clustered growth pattern of SCLC-A, SCLC-N tumor cells typically grow in loosely adherent monolayers. SCLC-P, comprising approximately 10% of cases, represents a non-neuroendocrine subtype defined by expression of epithelial lineage genes such as E-cadherin, SOX9, ASCL2, and IGF1R, often accompanied by MYC activation. SCLC-Y, initially described as a rare non-neuroendocrine subtype present in 2–10% of cases, has been associated with immune-related gene signatures, including interferon-gamma (IFN-γ) response pathways, T-cell receptor signaling, and HLA expression. It is also linked to activation of NOTCH signaling, mTOR, and PLK1 pathways, as well as retention of functional RB1, suggesting a more differentiated state with increased chemoresistance (47, 48). However, the concept of SCLC-Y as a distinct and stable molecular subtype has been fundamentally challenged. Critical re-evaluation reveals that several cell lines once classified as SCLC-Y often carry pathogenic SMARCA4 mutations and exhibit molecular and pathological features consistent with SMARCA4-deficient undifferentiated tumors rather than small cell lung cancer (49, 50). Therefore, YAP1 expression is increasingly viewed as a sporadic or transitional phenotype, not a subtype-defining transcription factor (51). Following this re-evaluation, the SCLC-I (inflamed) subtype is recognized as the principal non-neuroendocrine category in contemporary classifications, incorporating the immune-enriched features previously attributed to SCLC-Y (35). SCLC-I, representing approximately 15–17% of cases, is defined by low expression of ASCL1, NEUROD1, and POU2F3, along with a prominent inflammatory gene signature featuring high BTK expression. This subtype exhibits non-neuroendocrine and mesenchymal characteristics within an inflamed tumor microenvironment.

Clinical outcomes vary significantly across subtypes, reflecting their distinct molecular and immune profiles. Analysis of a cohort of patients with resectable SCLC treated with surgery and standard adjuvant therapy (without immunotherapy) has revealed distinct prognostic associations across molecular subtypes. SCLC-A is associated with a 5-year OS rate of 61.9%, indicating a moderate prognosis. SCLC-N shows a slightly improved outcome, with a 5-year OS of 69.3%. SCLC-P has the worst prognosis, with a 5-year OS of 41.7%, potentially due to its aggressive epithelial phenotype and strong association with smoking. In contrast, SCLC-I demonstrates the most favorable prognosis, with a 5-year OS of 85.7%, likely attributable to its inflamed tumor microenvironment and enhanced responsiveness to immunotherapy (52). Data on SCLC-Y remain limited owing to its rarity, but its immune-activated phenotype suggests a potentially better prognosis than SCLC-A, though not as favorable as SCLC-I.

Collectively, this transcription factor-based classification system refines the earlier binary distinction and provides a mechanistic framework for understanding the molecular heterogeneity of SCLC.

### 2.2 Subtype-specific immune landscapes in SCLC

The transcription factor-defined subtypes of small cell lung cancer (SCLC) exhibit distinct interactions with the immune microenvironment, which may critically influence therapeutic responses. Single-cell transcriptomic analyses reveal that SCLC-A and SCLC-N, both characterized by neuroendocrine features, predominantly display an “immune cold” phenotype, marked by minimal immune cell infiltration (53). Among these, SCLC-A demonstrates moderate immune activity, with approximately 26% of cases exhibiting an inflamed phenotype characterized by increased T-cell infiltration and interferon-γ signaling. This suggests a subgroup with potential sensitivity to immunotherapy. In contrast, SCLC-N is consistently associated with a more profoundly immunosuppressive microenvironment, with only 11% of cases showing inflammatory signatures, underscoring its general resistance to immune-based treatments.

Non-neuroendocrine subtypes, particularly SCLC-P and SCLC-Y, represent a biologically distinct subset with unique immunological profiles. Transcriptomic studies consistently associate these subtypes with elevated inflammatory gene expression and increased immune infiltration, classifying them as predominantly “immune hot” tumors within the SCLC subtypes (53). This inflamed phenotype is characterized by extensive infiltration of immune and stromal cells. However, it is critical to distinguish between infiltration and functional immune activity, as this microenvironment can also harbor functionally exhausted or senescent T cells and other suppressive cells. Thus, while immunologically distinct, this microenvironment does not guarantee sensitivity to immunotherapy.

The recently identified SCLC-I subtype is distinguished by a robust immune-active profile, with significantly elevated expression of CD8A and CD8B, indicative of enhanced cytotoxic T-cell infiltration compared to other subtypes. This finding has direct clinical implications. Retrospective analysis of the IMpower133 trial demonstrated that the survival benefit of first-line platinum-etoposide chemotherapy combined with atezolizumab was largely confined to patients with SCLC-I tumors when compared to all other cases combined (HR, 0.566; 95% CI, 0.321-0.998) (35). In contrast, SCLC-N maintains the most immunosuppressive microenvironment among all subtypes, providing a plausible explanation for the limited efficacy of immune checkpoint inhibitors in this subtype (54).

### 2.3 Key immunosuppressive cell populations

Building upon the subtype-specific immune landscapes, it is particularly notable that the predominant SCLC-A subtype largely exhibits an immune-cold phenotype. This underscores the importance of immunosuppressive cellular populations, which shape the tumor microenvironment and reinforce immune exclusion.

#### 2.3.1 Regulatory T cells and immune evasion

Regulatory T cells (Tregs) represent a specialized subset of T lymphocytes that play a pivotal role in enforcing immune tolerance within the small cell lung cancer (SCLC) microenvironment, acting as key mediators of tumor-induced immunosuppression. Defined by the master transcription factor Foxp3, Tregs originate through both thymic development (natural Tregs) and peripheral induction from conventional CD4^+^ T cells (55). While essential for maintaining immune homeostasis and preventing autoimmunity, their accumulation within the tumor microenvironment establishes a potent mechanism of immune evasion that promotes malignant progression (56). Within the complex immunosuppressive landscape of SCLC, Tregs are actively recruited to tumor sites, where they enforce tolerance to tumor antigens through multiple, non-redundant mechanisms. Elevated intratumoral Treg infiltration has been consistently associated with poor prognosis across various malignancies, underscoring their value as clinically relevant negative prognostic indicators (57–60).

Treg-mediated immunosuppression is governed by a complex network of signaling pathways, with transforming growth factor-β (TGF-β) serving as a central regulatory node. TGF-β not only drives the peripheral conversion of conventional CD4^+^ T cells into induced Tregs but also enhances the suppressive functionality of pre-existing Treg populations (61). Within tumors, Tregs secrete inhibitory cytokines such as IL-10 and TGF-β, which directly impair effector T cell functions (62). Their suppressive influence extends to CD8^+^ T cells, as shown in melanoma models where neutralization of surface-bound TGF-β restores cytotoxic activity (63). The immunosuppressive capacity of tumor-infiltrating Tregs is further amplified by the expression of glycoprotein A repetitions predominant (GARP), a receptor that stabilizes latent TGF-β on the Treg membrane, with GARP levels strongly correlating with impaired anti-tumor responses (64, 65). Moreover, Tregs engage in crosstalk with other immunosuppressive cell types, including dendritic cells (DCs). Tumor-altered DCs secrete TGF-β, which in turn promotes the differentiation of CD4^+^ T cells into Tregs, thereby establishing a self-reinforcing immunosuppressive feedback loop (66, 67). Collectively, these mechanisms constitute a robust barrier to effective anti-tumor immunity and contribute to resistance against both conventional therapies and immune checkpoint blockade.

Therapeutically, targeting Treg-mediated immunosuppression in SCLC holds significant promise. Despite their predominantly immunosuppressive role, emerging evidence reveals functional heterogeneity among Treg subsets, with certain populations capable of producing interferon-γ (IFN-γ), a cytokine that may enhance the efficacy of immune checkpoint inhibitors (68). This duality underscores the necessity for precision strategies that selectively abrogate tumor-promoting Treg functions while preserving their homeostatic regulatory roles. Given the prominent TGF-β signaling signature observed in many SCLC tumors, therapeutic interventions aimed at disrupting the TGF-β pathway may be particularly effective in alleviating immunosuppression and sensitizing tumors to immunotherapeutic approaches.

#### 2.3.2 Tumor-associated macrophages in SCLC progression

Tumor-associated macrophages (TAMs) are pivotal contributors to the immunosuppressive landscape of SCLC and play a significant role in disease progression. These cells exhibit substantial heterogeneity in both functional phenotypes and spatial distribution across tumor compartments. Malignant cells recruit TAMs through the secretion of cytokines and chemokines, while microenvironmental factors—such as hypoxia, growth factors, and reactive oxygen species—drive their polarization away from tumoricidal (M1-like) states toward pro-tumorigenic (M2-like) phenotypes (69, 70). This phenotypic shift is accompanied by reduced cytotoxic potential and diminished production of pro-inflammatory cytokines, thereby fostering an immunosuppressive tumor microenvironment.

The functional impact of TAMs on tumor progression is largely dictated by their polarization state, with M2-polarized macrophages exerting predominantly pro-tumorigenic effects in SCLC (71, 72). Immunohistochemical studies demonstrate that the majority of CD68^+^ TAMs in neuroendocrine-low (NE-low) SCLC tumors express the M2 marker CD163, indicating the establishment of an immunosuppressive milieu within tumor nests. M2 polarization is driven by IL-4 and IL-13 and is associated with the secretion of VEGF and TGF-β, which promote angiogenesis, stromal fibrosis, and immune suppression. Notably, the density of CD163^+^ M2 macrophages differs between primary tumors and lymph node metastases, with higher infiltration typically observed in primary lesions (69).

The spatial organization of TAMs critically influences their functional specialization. CD68^+^ macrophages are predominantly localized within stromal bands and necrotic regions, although in highly infiltrated tumors they may also infiltrate tumor nests. In contrast, CD163^+^ macrophages exhibit a ramified morphology and are primarily enriched in stromal compartments, with limited presence in tumor epithelial zones. This compartmentalized distribution suggests context-dependent functional roles, wherein TAM-derived factors support tumor cell proliferation, migration, metastatic dissemination, and the induction of angiogenesis (69). Elucidating these spatial and functional dynamics offers promising avenues for therapeutic strategies aimed at reprogramming or depleting pro-tumorigenic TAMs in SCLC.

#### 2.3.3 Myeloid-derived suppressor cells

Myeloid-derived suppressor cells (MDSCs) constitute a major immunosuppressive population within the SCLC microenvironment and act in concert with TAMs to facilitate immune evasion. Originating from bone marrow progenitors, MDSCs are mobilized by tumor-derived soluble factors to peripheral tissues and the tumor site, where they acquire potent immunosuppressive functions (73). Phenotypically, MDSCs are distinguished from neutrophils by reduced CD16 and CD62L expression and elevated levels of arginase-1 (Arg-1), CD66b, and CD11b. Two primary subsets predominate in cancer: monocytic MDSCs (M-MDSCs) characterized by CD11b^ki^, Ly6C^ki^, and Ly6G^lo^ expression, and polymorphonuclear MDSCs (PMN-MDSCs) displaying CD11b^ki^, Ly6G^ki^, and Ly6C^lo^ markers (74, 75).

Within the SCLC microenvironment, both MDSC subsets exhibit enhanced immunosuppressive activity compared to their peripheral counterparts. M-MDSCs exert suppression through the secretion of anti-inflammatory cytokines (e.g., IL-10, TGF-β), production of reactive oxygen species (ROS), expression of immune checkpoint ligands (e.g., programmed death-ligand 1, PD-L1), and activation of immunosuppressive metabolic enzymes such as inducible nitric oxide synthase (iNOS) and Arg-1. These mechanisms collectively inhibit effector T cell responses and promote angiogenesis, thereby facilitating tumor progression. Computational modeling further highlights their role in metastasis, indicating that inhibition of MDSC recruitment could reduce the probability of metastatic dissemination by up to 50% under conditions of low systemic MDSC levels (76).

The accumulation of MDSCs is driven by tumor-derived signals that act at different stages of their development. Growth factors such as G-CSF, M-CSF, and GM-CSF promote population expansion. Cytokines including IL-1, IL-4, IL-6, IL-13, and TNF enhance functional maturation. Chemokines such as IL-8, CCL2, and CXCL12 direct facilitate their mobilization into the tumor microenvironment (77). Once established within the tumor, MDSCs suppress both innate and adaptive immunity. Particularly noteworthy is their profound influence on natural killer (NK) cells, with mathematical modeling revealing that MDSC-mediated inhibition of NK cells has a greater impact on tumor outcomes than direct effects on tumor growth rates.

Therapeutic approaches targeting MDSCs are being explored to overcome immunosuppression in SCLC and encompass several strategies. These include depleting MDSCs or reducing their accumulation, for which, in preclinical lung cancer models, the combination of gemcitabine with a superoxide dismutase mimetic has been shown to target MDSCs and reduce tumor growth (78). Another strategy aims to inhibit MDSC immunosuppressive function, reprogram their metabolism, or induce their differentiation into non-suppressive myeloid lineages. Notably, the differentiation agent all-trans retinoic acid (ATRA), when combined with a p53 vaccine in a clinical study of ES-SCLC patients, significantly reduced MDSC levels and enhanced vaccine-induced anti-tumor immunity (79). The efficacy of these interventions appears context-dependent, with greater predicted benefit in tumors enriched with NK cells and limited impact in microenvironments dominated by cytotoxic T cells. Incorporating MDSC-related parameters into predictive models has been shown to improve the accuracy of tumor outcome prediction from 63% to 82% (76), highlighting their prognostic relevance and therapeutic potential in SCLC.

## 3 Emerging therapeutic approaches for SCLC

### 3.1 Immune checkpoint inhibitors in SCLC

Building on insights into the immune landscape of SCLC, recent therapeutic advances have focused on strategies that restore antitumor immunity, with immune checkpoint inhibitors playing a central role in this progress (80).

#### 3.1.1 PD-1/PD-L1 inhibitors

The programmed cell death protein 1 (PD-1)/PD-L1 pathway is a key immune regulatory mechanism and a major target in cancer immunotherapy (81). Within the tumor microenvironment, PD-L1 binds to PD-1 receptor on T cells, suppressing their cytotoxic activity and enabling immune escape (82, 83). At the molecular level, engagement of PD-1 by PD-L1 or PD-L2 induces phosphorylation of the immunoreceptor tyrosine-based switch motif (ITSM) by Src family kinases. This recruits the phosphatase SHP2, which inhibits signaling molecules such as ZAP70 and PLCγ1, thereby blocking T-cell receptor signaling and reducing T-cell function. SHP2 also interferes with the RAS/MEK/ERK and PI3K pathways, further restricting T cell activation and proliferation (84).

Monoclonal antibodies targeting PD-1 or PD-L1 restore T cell function by preventing this inhibitory interaction. In SCLC, these agents have shown clinical benefit, particularly in combination with standard platinum-etoposide chemotherapy.

Two pivotal phase III trials have established PD-L1 inhibitors as first-line therapy for ES-SCLC. The IMpower133 trial evaluated atezolizumab plus carboplatin-etoposide, showing a significant improvement in OS compared with chemotherapy alone (median OS: 12.3 vs. 10.3 months; HR, 0.70; 95% CI, 0.54 to 0.91; p value = 0.0069). The survival benefit was sustained at 18 months (34.0% vs. 21.0%) (85). Notably, efficacy was independent of PD-L1 expression and blood-based tumor mutational burden, indicating that these biomarkers lack predictive value in ES-SCLC (86).

The CASPIAN trial further validated this approach, showing that durvalumab combined with platinum–etoposide chemotherapy significantly prolonged OS compared with chemotherapy alone (median OS: 13.0 vs. 10.3 months; HR 0.73; 95% CI 0.59-0.91; p value = 0.0047) (15). Long-term follow-up revealed three-year survival rates of 17.6% with durvalumab versus 5.8% with chemotherapy. The benefit was consistent across subgroups, including patients receiving either cisplatin or carboplatin (87).

The ADRIATIC trial extended the use of durvalumab to limited-stage SCLC (LS-SCLC). As consolidation therapy following concurrent chemoradiotherapy, durvalumab significantly improved OS (median OS: 55.9 vs. 33.4 months; HR 0.73; 95% CI 0.54-0.98; p value 0.0104), establishing a new standard of care for LS-SCLC (88). Both the CASPIAN and ADRIATIC trials demonstrated consistent efficacy across patient subgroups, with ADRIATIC suggesting greater survival gains in earlier-stage disease.

The ASTRUM-005 trial marked a significant advancement by demonstrating the efficacy of serplulimab, the first anti-PD-1 monoclonal antibody approved for ES-SCLC. When combined with carboplatin and etoposide, serplulimab significantly prolonged OS compared with chemotherapy alone (median OS: 15.4 vs. 10.9 months; HR 0.63; 95% CI 0.49-0.82; p value 0.0005) (89). The two-year survival rate was markedly higher with serplulimab (43.1%) than with chemotherapy (7.9%), indicating a substantial long-term benefit. Similar to IMpower133 and CASPIAN, ASTRUM-005 showed consistent efficacy across subgroups. However, its use of a PD-1 inhibitor and superior median OS distinguish it from PD-L1-targeted regimens (90).

Additional PD-1/PD-L1 inhibitors developed in China have expanded treatment options for ES-SCLC. The CAPSTONE-1 trial demonstrated that adebrelimab, a PD-L1 inhibitor, combined with chemotherapy improved OS (median OS: 15.3 vs. 12.8 months; HR 0.72; 95% CI 0.60-0.87) (91, 92). Tislelizumab (RATIONALE-312) and toripalimab (EXTENTORCH), both PD-1 inhibitors, achieved median OS of 15.6 and 14.6 months, respectively, compared with 13.5 and 13.3 months in the chemotherapy arms (93, 94). These agents, approved in China between 2021 and 2023, underscore the growing importance of PD-1 inhibitors in the management of ES-SCLC.

Collectively, these findings led to regulatory approvals: atezolizumab was approved by the FDA in March 2019, durvalumab in March 2020 for ES-SCLC and in 2024 for LS-SCLC, and serplulimab was approved by the China NMPA in January 2023, with subsequent approvals in the European Union (February 2025) and the United Kingdom (June 2025) for first-line treatment of ES-SCLC. Adebrelimab, tislelizumab, and toripalimab received NMPA approval for ES-SCLC between 2021 and 2023. Despite variations in trial design—including choice of platinum agent and number of chemotherapy cycles—the control arms across studies reported similar median OS (10.3–10.9 months), supporting cross-trial comparability. Real-world evidence further corroborates these results, with prolonged survival observed in patients treated with atezolizumab-based regimens (9). A comprehensive summary of currently approved PD-1/PD-L1 inhibitors for SCLC is provided in **Table 2**.

**Table 2:** Summary of PD-1/PD-L1 antibodies approved for first-line treatment of ES-SCLC.

Drug name	Target	Developer	Key trial	Indication	Trial arms & comparator	Approval regions & dates	Median OS (vs. chemo)	Median PFS (vs. chemo)	Safety (common ≥grade 3 AEs, %)
Atezolizumab	PD-L1	Roche	IMpower133	ES-SCLC First-Line	Atezolizumab+carboplatin/etoposide Placebo+carboplatin/etoposide	US: 2019/3; EU: 2019/9; China: 2020	12.3 vs. 10.3; HR 0.70 (0.54-0.91);	5.2 vs. 4.3; HR 0.77 (0.62-0.96)	Anemia (12%), Neutropenia (14%), Immune-related AEs (e.g., hypothyroidism 5%)
Durvalumab	PD-L1	AstraZeneca	CASPIAN	ES-SCLC First-Line	Durvalumab + platinum/etoposide Platinum/etoposide	US: 2020/3; EU: 2020/11; China: 2021/12	13.0 vs. 10.3; HR 0.73 (0.59-0.91);	5.1 vs. 5.4; HR 0.78 (0.65-0.94)	Neutropenia (24%), Anemia (9%), Immune-related AEs (e.g., rash 10%)
Serplulimab	PD-1	Henlius	ASTRUM-005	ES-SCLC First-Line	Serplulimab+carboplatin/etoposide Placebo+carboplatin/etoposide	China: 2023/1; EU: 2025/2; UK: 2025/6	15.4 vs. 10.9; HR 0.63 (0.49-0.82);	5.7 vs. 4.3; HR 0.48 (0.38-0.59)	Neutropenia (37%), Anemia (15%), Immune-related AEs (e.g., capillary proliferation 5%)
Adebrelimab	PD-L1	Hengrui	CAPSTONE-1	ES-SCLC First-Line	Adebrelimab+ carboplatin/etoposide Placebo+carboplatin/etoposide	China: 2023/3	15.3 vs. 12.8; HR 0.72 (0.60-0.87)	5.8 vs. 5.6; HR 0.67 (0.54-0.83)	Anemia (14%), Neutropenia (19%), Immune-related AEs (e.g., hypothyroidism 4%)
Tislelizumab	PD-1	BeiGene	RATIONALE-312	ES-SCLC First-Line	Tislelizumab+platinum/etoposide Placebo+platinum/etoposide	China: 2024/6	15.5 vs. 13.5; HR 0.66 (0.54-0.80)	4.7 vs. 4.3; HR 0.64 (0.52-0.78)	Neutropenia (30%), Anemia (12%), Immune-related AEs (e.g., rash 8%)
Toripalimab	PD-1	Junshi	EXTENTORCH	ES-SCLC First-Line	Toripalimab+platinum/etoposide Placebo+platinum/etoposide	China: 2024/6	14.6 vs. 13.3; HR 0.79 (0.65-0.97)	5.8 vs. 5.6; HR 0.67 (0.54-0.82)	Anemia (10%), Neutropenia (18%), Immune-related AEs (e.g., fatigue 5%)

ES-SCLC, Extensive-stage small cell lung cancer; OS, Overall survival; PFS, Progression-free survival; HR, Harzard ratio; AE, adverse event.

In summary, the IMpower133, CASPIAN, ADRIATIC, and ASTRUM-005 represent major advances in SCLC. They established immunochemotherapy and consolidation immunotherapy as new standards of care. The introduction of serplulimab expands treatment options for ES-SCLC. However, direct comparative evidence on the relative efficacy of PD-1 versus PD-L1 inhibitors in this setting remains limited and does not support an advantage for either class. A recent real-world study directly comparing first-line chemoimmunotherapy regimens provides relevant insights. In this analysis of 322 ES-SCLC patients, the median PFS was 6.9 months for the PD-1 inhibitor serplulimab, compared to 7.63 months for atezolizumab, 7.43 months for durvalumab, and 7.4 months for adebrelimab (all PD-L1 inhibitors), with no statistically significant differences observed between serplulimab and any of the PD-L1 inhibitors (all p value > 0.05). For OS, the median was 15.0 months for serplulimab, versus 17.2 months for atezolizumab, 17.6 months for durvalumab, and 23.2 months for adebrelimab. Critically, while serplulimab’s OS was not significantly different from atezolizumab (p = 0.61) or durvalumab (p = 0.23), it was significantly shorter than that of the PD-L1 inhibitor adebrelimab (p = 0.029) (95). These results underscore the complexity of cross-class comparisons and indicate that therapeutic efficacy may vary significantly within the same inhibitor class, rather than being defined solely by targeting PD-1 versus PD-L1. Ongoing studies, such as KEYLYNK-013, which evaluates pembrolizumab in combination with chemoradiotherapy for LS-SCLC, aim to further refine and extend these strategies (96). While the benefit of checkpoint inhibition remains less pronounced than in some other cancers, these trials transformed the therapeutic landscape of SCLC and provided a foundation for further innovation in immunotherapy.

#### 3.1.2 CTLA-4 inhibitors

Cytotoxic T-lymphocyte-associated antigen 4 (CTLA-4) inhibitors constitute another class of immune checkpoint inhibitors that modulate antitumor immunity. Monoclonal antibodies targeting CTLA-4, such as ipilimumab and tremelimumab, block this inhibitory pathway to enhance T-cell activation and antitumor responses (84).

In SCLC, clinical outcomes with CTLA-4 inhibitors have been inconsistent. A phase III trial evaluating ipilimumab in combination with first-line chemotherapy for newly diagnosed ES-SCLC failed to demonstrate an improvement in OS compared with chemotherapy plus placebo (median OS: 11.0 vs. 10.9 months). Furthermore, the ipilimumab arm was associated with higher rates of treatment discontinuation (18% vs. 2%) and increased incidence of immune-mediated adverse events (97).

While monotherapy with CTLA-4 inhibitors has shown no meaningful efficacy in SCLC, dual blockade of CTLA-4 and PD-1 has yielded more promising results. These checkpoints regulate immune responses through distinct mechanisms: CTLA-4 primarily modulates early T-cell activation in lymphoid organs and enhances regulatory T-cell (Treg)-mediated suppression of dendritic cells, whereas PD-1 predominantly inhibits effector T-cell and natural killer (NK) cell function in peripheral tissues (98). Given their complementary roles, dual inhibition may elicit a more robust antitumor immune response than either agent alone (99).

This hypothesis was supported by a multicenter phase I/II study in patients with advanced SCLC (CheckMate-032) who had progressed after platinum-based chemotherapy. The combination of nivolumab (anti-PD-1) and ipilimumab demonstrated improved efficacy over nivolumab monotherapy in patients with relapsed SCLC. The confirmed objective response rate (ORR) was 10% (95% CI 5–18, n=98) with nivolumab monotherapy, compared to 23% (95% CI 13–36, n=61) with nivolumab (1 mg/kg) plus ipilimumab (3 mg/kg). The combination strategy also showed improved survival outcomes compared to monotherapy, with a median OS of 7.7 months (95% CI 3.6–18.0) and a 1-year OS rate of 43% (95% CI 30–56) versus 4.4 months (95% CI 3.0–9.3) and 33% (95% CI 22–45), respectively. However, the initial promising activity observed in CheckMate-032 did not translate into a statistically significant overall survival benefit in the subsequent Phase III CheckMate-451 trial, which evaluated these regimens as maintenance therapy after first-line chemotherapy. In CheckMate-451, the median OS was 9.2 months (95% CI, 8.2 to 10.2) for nivolumab plus ipilimumab, 10.4 months (95% CI, 9.5 to 12.1) for nivolumab monotherapy, and 9.6 months (95% CI, 8.2 to 11.0) for placebo. The primary endpoint of OS for the combination versus placebo was not met (HR, 0.92; 95% CI, 0.75–1.12) (100). These findings suggest that dual checkpoint inhibition may have a role in SCLC, although the magnitude of benefit is less pronounced than that observed in NSCLC (101).

However, the enhanced efficacy of combination therapy is accompanied by increased toxicity. Dual checkpoint blockade is associated with a higher risk of immune-related adverse events (irAEs) compared with monotherapy, necessitating vigilant monitoring and proactive management (99). It is important to note that dual checkpoint blockade regimens are not currently approved for the treatment of SCLC.

### 3.2 Delta-like ligand 3-targeted therapies

#### 3.2.1 Antibody-drug conjugates

The development of antibody-drug conjugates (ADCs) targeting Delta-like ligand 3 (DLL3) represents a significant advance toward precision therapy in SCLC. DLL3, an inhibitory ligand of the Notch signaling pathway, exhibits highly selective tumor expression, being present on the surface of approximately 85% of SCLC cells. In contrast, in normal tissues, DLL3 is primarily localized intracellularly and restricted to specific cell types, including neurons, pancreatic islet cells, and pituitary cells (23, 102). This distinct expression pattern provides an opportunity for tumor-selective targeting.

Rovalpituzumab tesirine (Rova-T), the first DLL3-targeted ADC, consists of a DLL3-specific IgG1 antibody linked to a DNA cross-linking pyrrolobenzodiazepine (PBD) dimer via a protease-cleavable linker. Preclinical studies demonstrated durable antitumor activity, including efficacy against platinum-resistant models. Early-phase clinical trials showed promise, particularly in patients with high DLL3 expression (103). In a phase I trial, an objective response rate of 35% was observed in patients with DLL3-high tumors (≥50% DLL3-positive cells), whereas no responses were reported in DLL3-low tumors (104). However, subsequent studies failed to confirm these encouraging results. The phase II TRINITY trial reported a lower ORR of 18%, and the phase III TAHOE trial, which compared Rova-T with topotecan, was terminated early due to inferior OS in the Rova-T arm.

The clinical failure of Rova-T was largely attributed to a narrow therapeutic window and substantial toxicity, including grade 4 thrombocytopenia, hepatotoxicity, and delayed pleural or pericardial effusions. These adverse events were largely attributed to the extreme potency of PBD dimer payload and the instability of the protease-cleavable linker, which contributed to premature payload release and “on-target, off-tumor” toxicity (104, 105). These outcomes underscore the difficulties in balancing potent cytotoxic payloads with acceptable safety profiles. In addition, resistance mechanisms have also been identified, including loss-of-function mutations in NOTCH1 that reduce dependency on DLL3, as well as phenotypic shifts from DLL3-high to DLL3-low states, reflecting the adaptive plasticity of SCLC under therapeutic pressure.

These failures provided critical lessons for the rational design of next-generation DLL3-targeted ADCs. Newer agents, including DB-1314 and ZL-1310, replace PBD dimer with topoisomerase I inhibitors, which offer a more favorable balance between potency and tolerability. Optimization of linker technology have furtherenhanced systemic stability and reduced premature payload release. DB-1314, for example, conjugates a humanized anti-DLL3 antibody to a topoisomerase I inhibitor via a peptidyl linker. Preclinical studies indicate that DB-1314 induces tumor cell killing through direct cytotoxicity, bystander effects, and antibody-dependent cellular cytotoxicity (ADCC), while exhibiting favorable pharmacokinetic and tolerability profiles in animal models (102). ZL-1310 has demonstrated particularly encouraging clinical activity in patients with ES-SCLC who progressed on or after platinum-based chemotherapy. Among patients treated at the recommended phase 2 dose (1.6 mg/kg) as second-line therapy (n = 19), the ORR reached 68%. Notably, substantial efficacy was also observed in clinically challenging subgroups, including an 80% ORR in patients with untreated baseline brain metastases and responses in patients previously treated with tarlatamab. These findings extend the clinical experience gained from Rova-T and provide clinical validation of the hypothesis that optimizing payload selection and linker design can substantially widen the therapeutic window.

Beyond ADCs, DLL3-targeted radioimmunotherapy, such as Lu-177-conjugated antibodies, is under investigation as a precision radiotherapeutic approach (106, 107).

Although DLL3-targeted ADCs have encountered setbacks, ongoing optimization of linker stability, payload selection, and drug-to-antibody ratios may enhance their therapeutic efficacy. Combination strategies, particularly with immune checkpoint inhibitors, may further improve efficacy by promoting immunogenic cell death and augmenting antitumor immunity. With increasing understanding of DLL3 biology and SCLC heterogeneity, next-generation ADCs may yet deliver meaningful clinical advances in this aggressive malignancy.

#### 3.2.2 Bispecific T-cell engagers

Bispecific T-cell engagers (BiTEs) represent a novel class of DLL3-targeted therapies that leverage immune effector mechanisms. BiTEs typically consist of two single-chain variable fragments (scFvs)—one binding DLL3 on tumor cells and the other engaging CD3 on T cells—often fused to an Fc domain to prolong half-life. By physically linking T cells to tumor cells, BiTEs facilitate major histocompatibility complex class I (MHC-I)-independent T-cell activation, resulting in perforin- and granzyme-mediated tumor lysis. This mechanism overcomes the low MHC-I expression commonly observed in SCLC, a key limitation of conventional checkpoint inhibitors (107).

Tarlatamab (AMG757), the first clinically evaluated DLL3-targeted BiTE, demonstrated encouraging activity in heavily pretreated SCLC patients, including those refractory to prior immunotherapy. In the phase I DeLLphi-300 single-arm trial, tarlatamab achieved an ORR of 23.4%, with a median PFS of 3.7 months (95% CI 2.1-5.4) and median OS of 13.2 months (95% CI 10.5-NE) (108). The phase II DeLLphi-301 trial compared dosing regimens and found that a lower dose (10 mg every two weeks) yielded comparable efficacy to a higher dose (100 mg) (ORR: 40% vs. 32%; median PFS: 4.9 (95% CI 2.9-6.7) vs. 3.9 months (95% CI 2.6-4.4)) but with reduced incidence of cytokine release syndrome (CRS) (any grade: 51% vs. 61%; grade ≥3: 1% vs. 6%) (109). The results of the phase III DeLLphi-304 trial have recently been published. This pivotal study demonstrated that second-line tarlatamab significantly improved OS compared to standard chemotherapy in patients who progressed after platinum-based therapy (median OS: 13.6 vs. 8.3 months; HR 0.60; p value < 0.001), with a lower incidence of grade ≥3 adverse events (54% vs. 80%) (110). This landmark study establishes tarlatamab as a new standard of care in this setting. Concurrently, the DeLLphi-306 trial is assessing tarlatamab as adjuvant therapy in LS-SCLC patients who remain disease-free after chemoradiotherapy, potentially expanding the role of immunotherapy to earlier disease stages. Preliminary data also suggest intracranial activity (111).

Looking beyond monotherapy, combination strategies are being explored to further enhance outcomes. The phase Ib DeLLphi-303 study evaluated tarlatamab in combination with a PD-L1 inhibitor (atezolizumab or durvalumab) as first-line maintenance therapy for extensive-stage SCLC. This approach is mechanistically rational, as the direct T-cell engagement by tarlatamab may synergize with immune checkpoint blockade. Early results are promising, with an objective response rate of 24% and a median duration of response of 16.6 months among 88 patients. The median OS was 25.3 months, with a 12-month OS rate of 82% (112). This sustained efficacy and notable survival signal in the maintenance setting highlight a promising future direction for DLL3-targeted therapy in earlier lines of treatment.

Several other DLL3-targeted BiTEs are in clinical development. BI764532 demonstrated a partial response rate of 25% across tumor types and 26% in SCLC patients at doses ≥90 μg/kg, with only 4% of patients discontinuing treatment due to adverse events (113). Other investigational agents include QLS31904, the trispecific engager HPN328 (engineered with albumin-binding capacity to extend half-life (114), and RO7616789, a dual-targeting construct that engages both DLL3 and CD137 (4-1BB) to enhance T-cell costimulation. These innovations aim to improve the efficacy, safety, and durability of BiTE-based therapies.

The emergence of BiTEs marks a potential shift in DLL3-targeted therapy. Unlike ADCs, BiTEs directly harness endogenous T cells and may induce immunological memory, offering the potential for sustained remissions (111). They also circumvent resistance mechanisms associated with ADCs, such as impaired lysosomal degradation or drug efflux. As development progresses, combining BiTEs with checkpoint inhibitors or other immunomodulatory agents may further enhance therapeutic outcomes in SCLC.

#### 3.2.3 CAR-T cell therapy approaches

Chimeric antigen receptor (CAR)-T cell therapy represents a durable form of immune redirection against DLL3-expressing tumors. CAR-T cells are genetically engineered to combine antigen recognition via an antibody-derived domain with T-cell activation signaling, enabling sustained cytotoxic activity following a single infusion. This approach bypasses the low MHC-I expression characteristic of SCLC and may offer longer-lasting responses than conventional therapies (115).

AMG119, the first DLL3-targeted CAR-T therapy, incorporates an anti-DLL3 binding domain with CD28 and 4-1BB costimulatory domains. Preclinical studies demonstrated potent activity against DLL3-expressing cells, even at low antigen density (<1,000 molecules per cell), along with robust cytokine release indicative of T-cell activation. In an early-phase trial in pretreated SCLC patients, AMG119 induced objective responses in 20% of participants, including a complete hepatic response, with manageable toxicity. Other DLL3-targeted CAR-T platforms, such as LB2102 and ALLO-213, are currently under investigation (107).

To overcome the immunosuppressive tumor microenvironment in SCLC, next-generation CAR-T cells engineered to secrete interleukin-18 (IL-18) have been developed. In preclinical models, DLL3-targeted IL-18-secreting CAR-T cells exhibited superior antitumor activity compared to conventional CAR-T cells, with reduced tumor growth and prolonged survival. Mechanistically, IL-18 enhanced T-cell persistence, reduced exhaustion, and amplified cytokine production. It also remodeled the tumor microenvironment by activating endogenous T cells and reprogramming myeloid cells toward an antitumor phenotype (115).

Combining CAR-T cells with checkpoint inhibitors offers another promising strategy. IL-18-secreting CAR-T cells targeting DLL3 demonstrated synergy with PD-1/PD-L1 blockade in xenograft models, highlighting the potential of combination therapy (115). Interestingly, this effect was restricted to IL-18 CAR-T cells, suggesting that cytokine secretion fundamentally alters therapeutic dynamics. Beyond T cells, CAR-engineered NK cells are also being explored, including NK-92-based constructs incorporating NKG2D and 2B4-CD3 signaling domains to enhance cytotoxicity (116).

Collectively, these advances highlight a growing shift toward cellular immunotherapy in SCLC. DLL3-targeted CAR-T and CAR-NK platforms exploit selective antigen expression while addressing key challenges in solid tumors, laying the groundwork for precision cellular therapies in this aggressive disease.

### 3.3 B7-H3 as an alternative therapeutic target

B7-H3 (CD276), a member of the B7 immune checkpoint family, has emerged as a promising therapeutic target due to its potent immunosuppressive effects in the tumor microenvironment. High B7-H3 expression inhibits T-cell proliferation and interferes with interferon-gamma (IFN-γ) production, thereby promoting immune evasion. Blockade of B7-H3 has been shown to reverse immunosuppression and enhance antitumor immunity across multiple cancers. In SCLC, elevated B7-H3 expression is associated with larger tumor burden and shorter OS, supporting its role as an independent prognostic marker (117).

Emerging evidence suggests that B7-H3 inhibition may act synergistically with other immunotherapies (118). A bispecific antibody-drug conjugate (BsADC) targeting both PD-L1 and B7-H3 demonstrated stronger immune activation than either monotherapy, resulting in increased CD8^+^ T-cell infiltration (119). This dual targeting induced direct tumor cell death and promoted immunogenic cell death, characterized by the release of damage-associated molecular patterns (DAMPs) that amplify adaptive immune responses. Flow cytometry analyses further revealed that B7-H3-targeted therapies upregulate CD69 on CD3^+^ T cells and CD86 on monocytes, indicating broad activation of immune cell populations (119). Collectively, these findings position B7-H3 inhibition as a promising strategy to convert immunologically “cold” tumors into “hot” tumors more responsive to immune attack.

### 3.4 Epigenetic regulators

Epigenetic regulators represent a promising class of therapeutic targets in SCLC. Lysine-specific histone demethylase 1 (LSD1; KDM1A) inhibitors, such as iadademstat (ORY-1001), have been shown to reactivate Notch signaling, suppress achaete-scute homolog 1 (ASCL1) activity, and inhibit tumor growth in preclinical SCLC models (120, 121). GSK2879552, another LSD1 inhibitor, has demonstrated potent antitumor effects, particularly when combined with PD-1 inhibitors (122). Another epigenetic target such as enhancer of zeste homolog 2 (EZH2), and the catalytic subunit of Polycomb Repressive Complex 2 (PRC2), is frequently overexpressed in SCLC and contributes to tumor progression by repressing the TGFβ-Smad-ASCL1 signaling pathway (123, 124).

## 4 Biomarkers for treatment selection of SCLC

### 4.1 Molecular subtypes as predictive biomarkers

#### 4.1.1 Subtype-specific therapeutic vulnerabilities

The distinct molecular subtypes of SCLC defined by specific transcription factor expression patterns, exhibit unique therapeutic vulnerabilities that can be leveraged for targeted treatment strategies. The SCLC-A subtype, characterized by high ASCL1 expression, displays elevated levels of MYCL and DLL3, rendering it particularly susceptible to DLL3-directed therapies (33). Furthermore, BCL-2, a direct transcriptional target of ASCL1, is overexpressed in this subtype, suggesting potential sensitivity to BCL-2 inhibitors (125). Notably, SCLC-A demonstrates a metabolic dependency on oxidative phosphorylation rather than glycolysis, a pathway commonly upregulated in cancers, thereby presenting an additional therapeutic opportunity (126).

The non-NE SCLC tumors, including SCLC-P and SCLC-Y subtypes, are associated with enhanced inflammatory gene signatures and increased immune cell infiltration, which may improve responsiveness to immune checkpoint inhibitors (53). Elevated POU2F3 mRNA expression has been correlated with increased sensitivity to lurbinectedin, a novel agent in SCLC therapy (127). Although most SCLC cell lines respond to lurbinectedin, those with high POU2F3 expression exhibit particularly favorable responses, supporting its role as a predictive biomarker for treatment selection (127).

Intratumoral heterogeneity and phenotypic plasticity present both challenges and opportunities in exploiting subtype-specific vulnerabilities. CD44 is highly expressed in non-NE populations, which are variably present across nearly all SCLC tumors, making it a viable target for combination therapies (53). Preclinical studies have demonstrated the efficacy of dual-targeting strategies, such as combining etoposide and cisplatin with silibinin, a CD44 promoter activity inhibitor, to simultaneously target neuroendocrine (NE) and non-NE malignant cell populations. Metabolic analyses further reveal that non-NE cells support NE cells through a metabolic coupling mechanism analogous to the astrocyte-neuron lactate shuttle, suggesting novel therapeutic approaches aimed at disrupting this cooperative interaction (126).

Collectively, mapping subtype-specific vulnerabilities and elucidating the functional interplay between cellular subpopulations provide a robust framework for advancing personalized therapeutic strategies in SCLC.

#### 4.1.2 MYC status

The MYC family of oncogenes plays a pivotal role in determining therapeutic response in SCLC. The SCLC-A subtype is marked by high MYCL expression alongside DLL3, forming a distinct molecular signature amenable to targeted intervention (103). In preclinical models, RRx-001—a small-molecule inhibitor of MYC that also downregulates CD47—has demonstrated promising antitumor activity by disrupting immune evasion via inhibition of the SIRPα-CD47 axis and repolarizing immunosuppressive M2 macrophages toward an antitumor M1 phenotype (128). This dual mechanism, targeting both oncogenic signaling and immune suppression, represents a novel strategy to overcome therapy resistance in MYC-driven SCLC.

MYC status also intersects with DNA damage response pathways. Inactivation of RB1 and TP53 abolishes key cell cycle checkpoints, increasing genomic instability and enhancing sensitivity to DNA-damaging agents (30). These findings suggest that combining MYC inhibition with DNA repair-targeted therapies may yield synergistic therapeutic effects.

### 4.2 Immune-related biomarkers

#### 4.2.1 Immune cell infiltration patterns

Recent molecular profiling has revealed distinct immune infiltration patterns that correlate with SCLC subtypes and clinical outcomes. Tumors with high neuroendocrine (NE-high) differentiation typically exhibit low densities of CD45+ immune cells and CD3+ T cells, consistent with an “immune-cold” phenotype that may contribute to their aggressiveness and poor response to immunotherapy. In contrast, NE-low tumors demonstrate higher immune cell infiltration, including substantial intratumoral T-cell presence, aligning with an “immune-hot” phenotype (69). This divergence suggests that NE-low tumors may be more responsive to immune checkpoint blockade, despite their typically immunosuppressive microenvironment.

Importantly, immune infiltration patterns may transcend conventional molecular subtyping. A subset of NE-high tumors exhibits immune-hot features with unique gene expression profiles distinct from classical NE-low tumors. Conversely, some NE-low tumors display immune-cold characteristics with molecular signatures differing from typical NE-high tumors (69). These observations underscore that molecular subtype alone is insufficient to capture the full complexity of immune heterogeneity. An integrated assessment incorporating both tumor-intrinsic features and the immune microenvironment may enable more accurate patient stratification for immunotherapy.

#### 4.2.2 PD-L1 expression

In contrast to other solid tumors where PD-L1 expression serves as a reliable predictive biomarker for response to immune checkpoint inhibitors, its utility in SCLC remains uncertain. Multiple clinical trials have consistently shown no significant correlation between PD-L1 expression and clinical benefit from immunotherapy in SCLC patients. This discrepancy highlights fundamental differences in the immune landscape of SCLC compared to other malignancies.

Evidence from landmark immunotherapy trials in ES-SCLC supports this observation. In the IMpower133 trial, which evaluated atezolizumab in combination with chemotherapy, exploratory analyses indicated clinical benefit regardless of PD-L1 immunohistochemistry status. With extended follow-up of 22.9 months, efficacy outcomes were comparable across PD-L1 expression subgroups (85). Similarly, in the KEYNOTE-604 trial assessing pembrolizumab plus chemotherapy, PFS and OS were similar in patients with PD-L1-positive and PD-L1-negative tumors (129).

The lack of predictive value may stem from complex regulation of PD-L1 within the SCLC microenvironment (130). Moreover, the high mutational burden characteristic of SCLC may drive robust T-cell activation independent of PD-L1 expression (8), potentially explaining the observed immunotherapy benefit across PD-L1 expression levels. Collectively, these findings indicate that while PD-1/PD-L1 signaling contributes to immune evasion, PD-L1 expression alone is inadequate for predicting immunotherapy response in SCLC.

#### 4.2.3 Tumor mutational burden

Tumor mutational burden (TMB) is a well-established biomarker for immunotherapy response in various cancers. SCLC is notable for its exceptionally high TMB, largely attributable to tobacco-related mutagenesis (30). This high mutational load is expected to enhance tumor immunogenicity through the generation of abundant neoantigens (131). However, clinical data reveal a paradox: despite comparable TMB levels, SCLC exhibits significantly lower response rates to immunotherapy than non-small cell lung cancer (NSCLC). This discrepancy can be attributed to differences in the immune microenvironment. SCLC tumors often display a restricted T-cell receptor (TCR) repertoire and reduced immune infiltration relative to NSCLC, impairing effective recognition of neoantigens despite high antigenic burden (132–134). These findings suggest that factors such as defective antigen presentation or unique immune evasion mechanisms in SCLC critically modulate therapeutic response.

Emerging evidence indicates that TMB may yield greater predictive power when integrated with other genomic and immune features. The “Gun-Bullet” model posits that both high TMB (bullets) and efficient antigen presentation via specific HLA alleles (gun) are necessary to elicit robust cytolytic activity and clinical benefit (85). Similarly, combining TMB assessment with measures of genomic instability may improve predictive accuracy, as genomic instability itself can hinder effective antitumor immunity (135).

The clinical utility of TMB in SCLC remains inconclusive, with variable results across studies. Future research should explore TMB in the context of SCLC molecular subtypes to refine its predictive value.

#### 4.2.4 Transcriptomic signatures

Transcriptomic profiles are emerging as potential predictors of immunotherapy response. For instance, SLFN11 expression was associated with improved outcomes in the S1929 trial, where patients with SLFN11-positive extensive-stage SCLC derived greater benefit from maintenance atezolizumab plus talazoparib compared to atezolizumab monotherapy (136). However, the clinical application of transcriptomic subtypes is complicated by the inherent plasticity of SCLC. These classifications often reflect transient transcriptional states rather than stable tumor identities (137). Coupled with significant intratumoral heterogeneity, this plasticity poses challenges for biomarker development and may facilitate therapeutic resistance through transcriptional state switching during disease progression or treatment. Robust predictive models will likely require integration of transcriptomic data with comprehensive immune contexture analysis.

### 4.3 Target expression as therapeutic indicators

DLL3 has emerged as a promising therapeutic target and biomarker in SCLC and other high-grade neuroendocrine carcinomas. Its overexpression is linked to tumor progression, poor prognosis, and dedifferentiation across multiple neuroendocrine malignancies (138). However, conventional immunohistochemistry (IHC) for DLL3 assessment faces several limitations, including the scarcity of recent tissue biopsies in aggressive diseases like SCLC, sampling bias due to intratumoral or inter-lesional heterogeneity, and high false-negative rates in histopathological evaluation (124). These challenges have catalyzed the development of immuno-positron emission tomography (immunoPET), enabling noninvasive, real-time, and quantitative assessment of DLL3 expression across all tumor sites (124).

Significant progress in DLL3-targeted imaging includes the development of 89Zr-labeled SC16 antibody-based radioimmunoconjugates, which effectively identify DLL3-expressing tumors in preclinical models. These agents exhibit uptake proportional to DLL3 expression, with greater accumulation in DLL3-high tumors compared to those with lower expression. Importantly, their performance has been validated across diverse models—including subcutaneous xenografts, orthotopic lung tumors, and metastatic lesions—with strong concordance between PET signals and bioluminescence imaging (124). Novel agents, such as [89Zr]-DFO-DLL3-scFv, further extend this approach to small cell neuroendocrine cancers beyond lung cancer, underscoring its broader applicability (139).

The clinical potential of DLL3-targeted immunoPET extends beyond tumor detection to predicting therapeutic response. Preclinical studies have demonstrated a strong correlation between PET signal intensity and response to DLL3-targeted therapies in patient-derived xenografts (124). This predictive capacity is particularly relevant in the context of acquired resistance, as tumors relapsing after DLL3-directed bispecific T-cell engager therapy frequently exhibit reduced DLL3 expression, implicating antigen loss as a resistance mechanism—especially in heterogeneous tumors. With first-in-human trials currently underway (140), DLL3-targeted immunoPET holds promise as a companion diagnostic tool to guide patient selection and monitor treatment response, potentially overcoming the limitations of static, tissue-based biomarker assessment and enabling precision medicine in this challenging disease.

### 4.4 Emerging biomarkers

Conventional biomarkers have shown limited utility in SCLC, prompting the exploration of novel approaches. Epigenetic biomarkers have gained prominence due to their central role in regulating antitumor immunity. Epigenetic mechanisms govern multiple aspects of immune function, including T-cell development, effector differentiation, and exhaustion. The use of epigenetic modifiers may remodel the immune microenvironment in SCLC and enhance responsiveness to immunotherapy (141). Epigenetic reprogramming influences not only CD8+ T-cell fate but also contributes to T-cell dysfunction and promotes the differentiation of myeloid-derived suppressor cells. Additionally, regulatory T cells exhibit distinct transcriptional and epigenetic profiles that reinforce immunosuppressive networks (142). These insights suggest that epigenetic landscapes may serve as more informative biomarkers than static genomic alterations.

In SCLC, traditional biomarkers such as PD-L1 expression and TMB have limited predictive value, reflecting the unique immunobiology of this disease. Emerging strategies that integrate molecular subtypes, immune contexture, and epigenetic regulation offer greater promise for guiding immunotherapy. Future biomarker development will likely necessitate multidimensional and dynamic assessments to enable truly personalized treatment approaches.

## 5 Conclusion

This review underscores the significant progress in elucidating the complex immunosuppressive microenvironment of SCLC and translating these insights into innovative therapeutic approaches. Immunotherapy has fundamentally transformed the treatment paradigm for SCLC, delivering clinically meaningful survival benefits after decades of therapeutic stagnation. Progress in molecular subtyping and immune profiling has uncovered distinct biological vulnerabilities, with DLL3-targeted therapies demonstrating notable efficacy, alongside the emergence of antibody-drug conjugates, bispecific T-cell engagers, and cellular immunotherapies.

Despite these advances, significant challenges persist. Conventional biomarkers, including PD-L1 expression and tumor mutational burden, exhibit limited predictive utility in SCLC, underscoring fundamental differences in the tumor immune microenvironment compared to other malignancies. The pronounced tumor heterogeneity, phenotypic plasticity, and highly immunosuppressive milieu of SCLC collectively impede the achievement of durable treatment responses. Additional practical obstacles, such as limited tissue availability, rapid disease progression, and restrictive clinical trial designs, further complicated the development and clinical implementation of robust biomarkers.

Future progress will require integrative strategies that combine molecular and immune profiling to guide therapy, rationally design combination regimens to overcome resistance mechanisms, and leverage non-invasive technologies such as functional imaging for real-time disease monitoring. Key research priorities include targeting the neuroendocrine-to-non-neuroendocrine transition, engineering multifunctional immune cell-based therapies, and reprogramming myeloid cells to remodel the immunosuppressive tumor microenvironment. Moreover, clinical trials must adopt more inclusive eligibility criteria to better represent the broader patient population. A key example is the underrepresentation of patients with brain metastases. Although brain metastases are present in approximately 15-20% of patients with SCLC at diagnosis (143), pivotal first-line immunotherapy Phase III trials (e.g., IMpower133, CASPIAN, CAPSTONE-1) typically enroll only patients without or with neurologically stable brain lesions (87, 90, 91, 144). This leads to a much lower proportion of such patients in trials (ranging from 2.2% to 13%) compared to their real-world prevalence, limiting the generalizability of findings to this common and prognostically challenging subgroup. Future trial designs should prospectively plan to include a more representative proportion of patients with brain metastases. Ultimately, precision immunotherapy approaches that dynamically adapt to the evolving biology of SCLC offer the most promising path toward improving outcomes in this aggressive malignancy.
